# Case Report: Neonatal Cholestasis as Early Manifestation of Primary Adrenal Insufficiency

**DOI:** 10.3389/fped.2021.767858

**Published:** 2021-11-11

**Authors:** Fabiola Di Dato, Donatella Capalbo, Rita Mirra, Francesca Del Vecchio Blanco, Mariacarolina Salerno, Raffaele Iorio

**Affiliations:** ^1^Section of Pediatrics, Department of Translational Medical Sciences, University of Naples Federico II, Naples, Italy; ^2^Department of Precision Medicine, University of Campania L. Vanvitelli, Naples, Italy

**Keywords:** cortisol, familial glucocorticoid deficiency (FGD), hypoglycemia, liver, jaundice

## Abstract

Neonatal cholestasis (NC) may be due to multiple surgical and non-surgical causes, some of which are potentially fatal. The list of potential causes of NC is long, and the systematic search for each of them is challenging in infants, especially when overt signs of underlying disease are lacking. Endocrinological diseases as causes of NC are rare and sometimes misdiagnosed. We report the case of an infant with prolonged cholestatic jaundice due to adrenal insufficiency suspected because of a single episode of hypoglycemia occurring at birth in the absence of clinical signs of adrenal impairment. Clinical exome analysis identified a new homozygous variant in MC2R gene as a putative responsible for familial glucocorticoid deficiency (FGD). Adrenal insufficiency should always be considered in all cholestatic infants, even in the absence of specific symptoms, since early recognition and treatment is essential to prevent life-threatening events.

## Introduction

Neonatal cholestasis (NC) is defined as an impairment in bile flow resulting in retention of bile substances, generally characterized by an increase in serum direct bilirubin and bile acid levels (1). It may be due to multiple surgical and non-surgical causes, some of which are potentially fatal. Clinical manifestations can range from initially paucisymptomatic forms, such as biliary atresia to severe conditions of clinical derangement such as galactosemia and other metabolic disorders (1). For some etiologies, early diagnosis is strongly recommended because specific and effective treatments are available. Unfortunately, as a result of the absence of significant clinical signs, some cholestatic infants can be misdiagnosed as affected by common and transient forms of unconjugated hyperbilirubinemia such as physiological or breastfeeding jaundice. Hence, NC guidelines recommend testing the conjugated serum bilirubin in all jaundiced breast-fed or formula-fed infants after 3 or 2 weeks of life, respectively (1). The list of potential causes of NC is long, and their systematic research is partly hampered by the difficulty in taking many blood samples from young infants, especially in the absence of overt signs of disease. Endocrinological diseases, such as hypopituitarism and congenital hypothyroidism, are well-known causes of NC (1). Several studies have shown that the pituitary–adrenal axis affects the synthesis of bile acids and that cortisol deficiency may have a role in inducing cholestasis (2–5). Primary adrenal insufficiency (PAI) is a potentially life-threatening disease characterized by impaired secretion of glucocorticoids eventually associated with mineralocorticoid and adrenal androgen deficiency or excess (6, 7). Although PAI is known to be a possible cause of NC, cholestasis has not been reported as its isolated clinical manifestation so far. In children, PAI is mainly due to inherited disorders of steroidogenesis, including familial glucocorticoid deficiency (FGD), which is characterized by isolated glucocorticoid deficiency (6). Here, we report the case of an infant with prolonged cholestatic jaundice due to PAI suspected because of a single episode of hypoglycemia occurring at birth in the absence of other clinical signs of adrenal insufficiency. A novel homozygous variant in MC2R gene was identified as a possible cause of FGD.

## Case Report

The patient was male, born to healthy unrelated parents after 40 weeks of gestation by cesarean section for early decelerations. Maternal viral markers for HIV, HBV, and HCV were negative. The mother was immune to toxoplasma, CMV, and rubella. Birth weight was 2.980 kg, appropriate for gestational age, length 51 cm, head circumference 36 cm. On the first day of life, he was admitted in the neonatal intensive care unit for hypoglycemia requiring early enteral feeding and a single glucose intravenous infusion with subsequent stabilization of blood glucose levels. Due to the presence of transient tachypnea, he received O_2_ supplementation for the first 48 h of life. During hospitalization, the neonate underwent transfontanellar and abdominal ultrasounds with normal results. Echocardiography showed muscular ventricular septal defect with mild left-to-right shunt without hemodynamic consequences. Complete blood count, coagulation, blood culture, and urinalysis were normal. Extended neonatal screening for metabolic diseases, performed according to regional guidelines (8), congenital hypothyroidism, and cystic fibrosis were negative. Due to the presence of jaundice with mild cholestasis since day 4, ursodeoxycholic acid therapy was started on the seventh day of life at a dose of 25 mg/kg per day. At the age of 28 days, the patient was referred to our pediatric liver unit to investigate cholestasis. At admission, the infant was healthy looking with normal weight and length on mixed breast- and formula milk feeding. On physical examination, there was a mild jaundice, a normal-sized liver of normal consistency, and colored stools. External genitalia were male with palpable testis. No cutaneous or mucosal hyperpigmentation was observed. Laboratory evaluation confirmed cholestasis with normal values of liver enzymes, prothrombin time, and albumin (Table 1). Biliary atresia was excluded not only due to normal colored stools but also because fasting abdominal ultrasound showed a normal-sized liver with preserved echostructure and absence of the following typical signs: triangular cord, abnormal gallbladder morphology and contractility, increased subcapsular liver flow, and splenic malformations. The following other causes of neonatal cholestasis were excluded: infections, cystic fibrosis, α-1-antitrypsin deficiency, Alagille syndrome, and progressive familial intrahepatic cholestasis. To investigate possible metabolic diseases, the dosage of serum acylcarnitines, serum amino acids, and urinary organic acids was carried out with normal results.

**Table 1:** Time-course of laboratory parameters and therapies performed in an infant with MC2R adrenal insufficiency.

Age–days	1	4	6	8	14	25	28	31	40	45	55	76	122	189	294
Na+ mEq/L							134	135	135	133	134	135	133	135	141
K+ mEq/L							5.8	5.6	5.2	6	5.7	5.2	5.5		4.9
Blood glucose mg/dl							87	51	60	55	72	87	111	99	91
Total Bilirubin mg/dl	3.79	13.88	13.1	11.73	9.38	7.37	8.04	7.56	6.11	4.79	0.73	0.52	0.29	0.57	0.55
Direct Bilirubin mg/dl	0.72	1.69	3	3.03	2.64	2.26	1.33	1.34	1.45	1.38		0.28			
AST U/L							47	43	54	43	68	43	40	48	46
ALT U/L							10	11	16	11	27	32	27	27	24
GGT U/L								194		176	158	67	26	15	16
Therapy				UDCA	UDCA	UDCA	UDCA	UDCA	UDCA HC	UDCA HC	UDCA HC FC	HC FC	HC FC	HC FC	HC FC

*UDCA, ursodeoxycholic acid; HC, hydrocortisone; FC, fludrocortisone*.

Since hypopituitarism is a cause of NC, although only a single episode of hypoglycemia occurred at birth, the main endocrine causes associated with NC were investigated, and blood glucose was monitored. Blood glucose levels remained normal even after prolonged fasting, while hormonal evaluation revealed undetectable levels of cortisol (<20 μg/dl) with very high levels of ACTH (5,549 pg/ml) suggestive of PAI (7). Adrenal precursors were all very low except for testosterone which was adequate to minipuberty. Renin and electrolytes were normal at first evaluation; mild transient hyperthyrotropinemia (TSH 11.404 μUI/ml, fT4 1.07 ng/dl) was also found, but not confirmed at a second evaluation.

The child was promptly treated with hydrocortisone (22 mg/m^2^/day) with early normalization of conjugated bilirubin values. Ursodeoxycholic acid therapy was withdrawn at the age of 2 months with no recurrence of cholestasis. During the follow-up, a trend toward progressive increase in renin levels (252.6 pg/ml, nv 2.52–35.82) was observed, and mineralocorticoid replacement therapy was introduced at 55 days of life.

As for PAI etiology, low-normal levels of 17-hydroxyprogesterone excluded congenital adrenal hyperplasia due to 21OH deficiency. Moreover, adrenal autoantibodies, very long-chain fatty acids, and adrenal ultrasound were also normal, thus, excluding autoimmunity, X-linked adrenoleukodistrophy, or adrenal hemorrhage. There were no complex or multisystem features suggesting a specific syndromic condition. Clinical exome sequencing including about 5,200 mendelian disease genes (constitutional custom panel, Agilent), was performed in trio (proband, father, mother) to identify the etiology of PAI. The analysis showed that in the proband, a previously unreported Thr33Lys homozygous variant in MC2R gene was potentially responsible for FGD. The Sanger sequencing confirmed the same variant in both the parents in a heterozygous state.

On the last observation, the patient was 9 months old, of general well-being, on stable therapy with hydrocortisone and fludrocortisone. His weight and length were appropriate for his age, and the serum bilirubin and electrolytes values were normal. Table 1 shows the time-course of the laboratory parameters during the entire observation period and the therapies performed.

## Discussion

In the neonatal period, the liver responds with cholestasis to numerous stimuli for which an impairment in bile flow can result from multiple entities including endocrinological disorders, which are rare and sometimes misdiagnosed (7, 9). It is reported that a fair number of infants with cholestasis due to endocrinopathy receive a delayed diagnosis because of mild clinical signs, and liver biopsy is sometimes performed before the diagnosis of endocrinopathy is established (10–13). According to the NC guidelines, endocrinological disorders have to be carefully sought in order to identify hypopituitarism or hypothyroidism as a cause of cholestasis (1), but little attention seems to be reserved to PAI.

PAI is a potentially life-threatening disorder requiring prompt recognition and treatment (7, 14). Since PAI often presents with non-specific symptoms, there is a significant delay in diagnosis resulting in increased morbidity and mortality, even more evident for FGD (11, 15). FGD is a group of rare conditions characterized by a lack of response to ACTH and the presence of isolated glucocorticoid deficiency. FGD usually presents in the early period of life with hypoglycemia, convulsions, prolonged jaundice, and marked skin hyperpigmentation (6, 11, 14). Several defects in MC2R, MRAP, MCM4, NNT, and TXNRD2 genes are related to FGD or related conditions, and MC2R variants cause about 25% of all cases of FGD (15, 16). In our patient, clinical exome analysis showed a homozygous variant in MC2R gene, potentially causative of FGD, never previously reported. It is to note that molecular analysis now enables rapid and affordable diagnosis for many causes of cholestasis whose identification is challenging with conventional tests (17).

Presentation of AI with cholestasis is limited only to the early infantile period, but in the cases reported so far, cholestasis was associated with extrahepatic manifestations. Beyond infancy, the hepatic manifestations due to AI include elevation of liver enzymes but not cholestasis (18). Infants with AI have been reported to show conjugated hyperbilirubinemia at a median age of 13–18 days, followed by raised transaminases 2–4 weeks later, while GGT concentration remained frequently normal or slightly elevated (10, 12, 13). Al Hussaini et al. in 2012 described four male infants with prolonged neonatal jaundice due to isolated cortisol deficiency (19). All were born from consanguineous parents with median age at observation of 10 weeks (range 6–12) and with mild hepatomegaly. Two of them received diagnosis of FGD, while the remaining two of isolated ACTH deficiency. In all these infants, recurrent hypoglycemia was detected, and the two infants with FGD also showed hyperpigmented skin (19). Different from these cases, our infant presented with early onset mild cholestasis in the absence of any other sign of AI such as recurrent hypoglycemia or hyperpigmented skin; moreover, the size of liver was normal. Furthermore, as for liver enzymes, Al Hussaini et al. observed elevated levels of aminotransferases with normal GGT levels (19), while in our case, GGT was elevated for sex and age, but other serum liver enzymes were normal probably due to the very early diagnosis.

Children with FGD usually respond very well to glucocorticoid replacement even if serum ACTH levels can remain high (14). In the study of Hussaini, cholestasis was resolved in 3 months (19). Differently, in our case, direct bilirubin levels normalized after 2 weeks of cortisol supplementation, while high ACTH levels persisted at the last observation in spite of an adequate hormone replacement.

Partial or complete deficiency of sex hormones such as testosterone, progesterone, and dehydroepiandrosterone sulfate was reported in several FGD patients (15, 20). Although associated mineralocorticoid insufficiency is very rare, transient salt loss can also occur (11, 14, 16). Renin and aldosterone were slightly elevated in our patient so that replacement therapy was added even in the absence of serum electrolyte abnormalities. Accordingly, it has been reported that patients with variants in the MC2R gene require temporary mineralocorticoid replacement (20, 21).

The main message of this case report is to encourage pediatricians to systematically research PAI in all cases of NC including paucisymptomatic patients and also in the absence of overt signs of AI. In fact, in our case, hypoglycemia was only transient, while the unexplained cholestasis was the main sign presented. It is to note that even after 6 h of fasting required for abdominal ultrasound, no hypoglycemia occurred in our patient. This could lead to the neglect of the single episode of hypoglycemia at birth. It is to note that while hypoglycemia associated with perinatal risk factors such as low gestational age and low birth weight may reasonably not be considered a warning sign, in the absence of risk factors (as in our case), hypoglycemia has to be valued.

The systematic search for endocrinological causes in all cholestatic infants raises the question on how far it is necessary to extend the search for possible etiologies in paucisymptomatic cholestatic infants. These considerations are also of value for the economic implications and the awareness that the NC can be transient in a variable number of cases (1, 22). As for transient cholestasis, it is an emerging subgroup among patients with neonatal cholestasis (23), and the guidelines do not provide specific recommendations (1). On the basis of our case, in all infants with unexplained cholestasis, cortisol serum levels should be tested.

In conclusion, this case of FGD, associated with a novel variant in MC2R gene, presenting only with NC and a single transient episode of hypoglycemia, should alert physicians to investigate the presence of PAI in order to favor prompt recognition and treatment replacement and avoid further invasive diagnostic tests including liver biopsy in any infant with unexplained cholestasis.

## Data Availability Statement

The original contributions presented in the study are included in the article/supplementary material, further inquiries can be directed to the corresponding author/s.

## Ethics Statement

Written informed consent was obtained from the minor(s)' legal guardian/next of kin for the publication of any potentially identifiable images or data included in this article.

## Author Contributions

FDD and RI: article conception. RM and FDVB: data collection. FDD, FDVB, MS, and DC: data analysis and interpretation. FDD and RM: drafting of the manuscript. RI, MS, and DC: critical revision of the manuscript. All authors approved the final version of the manuscript and agree to be accountable for the content of the work.

## Funding

Department of Translational Medical Science of University of Naples Federico II provides funds for open access publication fee.

## Conflict of Interest

The authors declare that the research was conducted in the absence of any commercial or financial relationships that could be construed as a potential conflict of interest.

## Publisher's Note

All claims expressed in this article are solely those of the authors and do not necessarily represent those of their affiliated organizations, or those of the publisher, the editors and the reviewers. Any product that may be evaluated in this article, or claim that may be made by its manufacturer, is not guaranteed or endorsed by the publisher.

## Abbreviations

NC: neonatal cholestasis
PAI: primary adrenal insufficiency
FGD: familial glucocorticoid deficiency.
